# 高效液相色谱-串联质谱法结合稳定同位素标记肽段同时测定稻米及其制品中3种过敏蛋白质

**DOI:** 10.3724/SP.J.1123.2021.06039

**Published:** 2021-12-08

**Authors:** Huan YANG, Zhaoyun CAO, Youning MA, Mingxue CHEN

**Affiliations:** 1.农业农村部稻米及制品监督检验测试中心, 中国水稻研究所, 浙江 杭州 310006; 1. Rice Product Quality Inspection and Supervision Center, Ministry of Agriculture and Rural Affairs, China National Rice Research Institute, Hangzhou 310006, China; 2.江西农业大学, 江西 南昌 330000; 2. Jiangxi Agricultural University, Nanchang 330000, China

**Keywords:** 液相色谱-串联质谱, 特异性肽段, 稳定同位素标记肽段, 酶解条件, 过敏蛋白质, 稻米, liquid chromatography-tandem mass spectrometry (LC-MS/MS), signature peptide, stable isotope-labeled peptide, digest condition, allergenic protein, rice

## Abstract

基于稳定同位素标记特征肽段和液相色谱-质谱联用仪建立稻米及制品中3种过敏蛋白质的同时定量方法。稻米及制品样品经盐溶液提取,赖氨酰基内切酶(Lys-C)和胰蛋白酶依次水解,C18-SD柱净化后,采用纳升高效液相色谱-线性离子阱-静电场轨道阱(NanoLC-LTQ-Orbitrap)采集和Protein Discovery软件鉴定,NCBI和Uniprot数据库的基本局部搜索比对工具(BLAST)筛选验证,最终获得表征稻米及制品中*α*-淀粉酶/胰蛋白酶抑制剂类蛋白质(seed allergenic protein RAG2, RAG2)、乙二醛酶Ⅰ活性蛋白(glyoxalase Ⅰ)和*α*-球蛋白(19 kDa globulin)3种过敏蛋白质的特异性肽段。3个特异性肽段经液相色谱梯度洗脱,在Poroshell色谱柱上实现完全分离,由三重四极杆质谱仪分析。实验通过优化多反应监测(MRM)质谱参数,比较不同溶剂体系、水解酶种类和酶量等酶解条件,结合内标法定量,实现对稻米及制品中3种蛋白质的绝对定量。实验结果表明,当酶解溶剂中含1 g/L十二烷基硫酸钠,采用Lys-C和胰蛋白酶组合消化策略,可有效提高3种蛋白质的酶切效率至65.7%~97.3%。该方法在1~200 nmol/L范围内线性关系良好,相关系数均大于0.9972, 3种蛋白质的检出限和定量限分别为3 mg/kg和10 mg/kg。3种蛋白质在空白稻米制品基质中3个水平下的加标回收率为80.6%~103.7%,日间和日内精密度均小于11.5%。该方法稳定性好,检测灵敏度高,操作简便,在分析各类稻米及制品中3种过敏蛋白质含量具有广泛的应用前景。

## Abstract (en)

Rice is an important cereal that is consumed as both an energy and protein source by a large proportion of the population worldwide. However, clinical studies have found that rice grains are responsible for cases of severe asthma, eczema, and atopic dermatitis in some adult patients. Several allergenic proteins have been identified and biochemically and immunochemically characterized from rice grains. These include *α*-amylase/trypsin inhibitors, glyoxalase Ⅰ, and *α*-globulin. In this study, we proposed an approach for the simultaneous quantification of three allergenic proteins in rice and its products, based on a stable isotope-labeled signature peptide standard and liquid chromatography-tandem mass spectrometry. Samples of rice and products were extracted by a salt solution, hydrolyzed by Lys-C and Trypsin, and purified by C18-SD. The linear ion trap-high resolution mass spectrometry (LTQ-Orbitrap) and Protein Discovery software were used to acquire and identify allergenic proteins in rice samples. In present study, three proteins including seed allergenic protein RAG2, glyoxalase Ⅰ, and 19 kDa globulin were identified. To establish a stable quantitative detection method, the signature peptides selected from the identified enzymatic hydrolysis peptides must have greater abundance and higher specificity as characteristic peptides. Three corresponding signature peptides in rice were screened based on the principles of previous study, and were validated through comparisons of the basic local alignment search tool (BLAST) with the NCBI and UniProt databases. The three signature peptides were successively eluted by liquid chromatography and separated on a Poroshell column. They were then detected by positive electrospray ionization (ESI^+^) in multiple reaction monitoring mode and quantified by an isotope dilution method. To achieve an improvement in the detection sensitivity and specificity, mass spectrometry parameters, such as the collision energy of three ion pairs of each peptide, were optimized. Three recombinant allergenic proteins and the winged stable isotope-labeled signature peptide standard were synthesized. These were then used to compare the effects of different enzymatic conditions, including hydrolysis solvents containing sodium dodecyl sulfate (SDS) with different contents, as well as the enzymes and their amounts, on the digestion efficiency. The data showed that the digestion efficiency of the three proteins could be improved to 65.7%-97.3% when 1 g/L of the SDS-containing hydrolysis solvent, and the combined digestion strategy of Lys-C and Trypsin, were adopted in the enzymatic process. These results indicate the following inferences: a small amount of SDS (1 g/L) in the enzymatic hydrolysis system is beneficial to complete protein denaturation, a Lys-C and Trypsin combined digestion strategy can complement the shortcomings of the two enzymes and improve the digestion efficiency, and the recoveries of the three proteins was not significantly increased by increasing the amount of enzyme when the ratio of protein to enzyme reached more than 20:1. The method displayed good linearity in the range of 1-200 nmol/L with the correlation coefficients greater than 0.9972. The limits of detection and limits of quantification of the three proteins were 3 mg/kg and 10 mg/kg, respectively. The average recoveries of the three proteins spiked at three levels in different matrices ranging between 80.6%-103.7%, with the intra-day and inter-day precision less than 11.5%. Due to its high stability, excellent sensitivity, and simple operation, this method presents a wide range of application prospects in the analysis of the three allergenic proteins in different rice and rice food products.

水稻(*Oryza sativa* L.)作为一种碳水化合物和蛋白质的重要来源,通常被认为是低致敏性作物,可作为一些谷类敏感患者的饮食替代品。但近年来临床研究发现,大米可导致一部分人产生过敏性皮肤炎、过敏性荨麻疹等症状,而过敏反应的发生和严重程度与过敏原种类、过敏原含量和摄入量息息相关^[1,2,3]^。因此,越来越多的研究致力于鉴定和降低大米中潜在的过敏蛋白质^[4,5,6,7]^。此外,为保护过敏消费者,美国、欧盟、日本、韩国相继出台政策,要求在食品标签上标注致敏食品成分的相关信息^[8,9,10]^,但因大米过敏蛋白质缺少有效的检测方法和阈值,导致这些政策无法适用于管理大米及其制品商品标签上相关过敏原含量的信息,提示过敏消费者自主规避。因此,开展快速测定大米中过敏蛋白质含量的方法研究,对于监管稻米制品生产过程和保护大米过敏患者具有重要意义。

目前,据文献报道在稻米中已鉴定到*α*-淀粉酶/胰蛋白酶抑制剂类蛋白质(seed allergenic protein RAG2, RAG2)、乙二醛酶Ⅰ活性蛋白(glyoxalase Ⅰ)、*α*-球蛋白(19 kDa globulin)和磷脂转移蛋白等多种过敏蛋白质^[11]^。传统的免疫亲和方法如基于抗体的免疫吸附试验是分析稻米中过敏蛋白质的主要方法,该方法操作简便,可商业化生产,具有低成本、高灵敏度等特点^[12,13]^。但同源性蛋白质之间可通过抗体发生交叉反应导致假阳性,且免疫试剂反应只能检测单一过敏原,无法实现同时定性定量检测多个蛋白质。基于质谱多反应监测(multiple reaction monitoring, MRM)的靶向蛋白质组学技术具有高灵敏度、高通量和高重复性等技术特点,可对复杂样品中多种靶蛋白进行准确定量,被视为靶向蛋白质绝对定量的重要手段^[14,15]^。尽管MRM质谱技术具有诸多优势,但该方法是基于“Bottom-Up”策略,通过分析酶解蛋白质产生的特异性肽段实现相应靶蛋白的绝对定量。多项研究发现,目标蛋白质的提取效果、酶解效率和杂质去除等步骤会严重影响方法准确度和重复性,对样品制备具有较高要求^[16,17]^。因此,有必要通过比较不同的提取条件,优化酶解过程,提高目标蛋白质特异性肽段的产出率,达到提高方法准确性的目的。此外,内标肽段的使用也可以减小分析过程中不稳定因素带来的定量误差,从而提高测定结果的准确度和可靠性。目前,基于MRM和同位素内标肽段的方法被广泛用于芝麻、水产品、巧克力和鱼等各类食品中过敏原的绝对定量^[18,19,20,21,22,23]^,但在稻米中过敏蛋白质定量分析的适用性研究鲜有报道。

本研究首先采用纳升高效液相色谱-线性离子阱-静电场轨道阱(NanoLC-LTQ-Orbitrap)高分辨质谱技术在稻米样品中鉴定出RAG2、乙二醛酶Ⅰ活性蛋白和*α*-球蛋白,并筛选得到3种蛋白质对应的特异性肽段。通过比较酶解体系、酶种类和酶量等条件,以侧翼同位素标记特征肽段为内标物,建立了同时定量分析稻米中3种主要过敏蛋白质的方法。该方法具有灵敏度高、线性范围广、重现性好等优点,可用于稻米及制品中过敏蛋白质的快速筛查和定量检测,为过敏蛋白质的标识提供技术支持。

## 1 实验部分

### 1.1 仪器、试剂与材料

EasynLC1000纳升液相色谱仪和LTQ-Orbitrap线性离子阱-静电场轨道阱组合高分辨质谱仪(ThermoFisher,美国); LC-20ADCR液相色谱仪(Shimadzu,日本); AB Sciex QTRAP 5500三重四极杆质谱仪(SCIEX,美国);脱盐固相萃取柱(3M Empore extraction disk cartridges, 7 mm/3 mL, C18-SD, 3M公司,美国);台式多用途高速离心机(ThermoFisher,美国);十万分之一天平(METTLER TOLEDO,美国); UV-2600紫外分光光度计(Shimadzu,日本); PHS-3C精密pH计(上海仪电科学仪器股份有限公司); Eppendorf ThermoMixer C恒温混匀振荡仪(Eppendorf,德国)。

3种蛋白质的特征肽段VVLVDNADFLK、DQVVYSLGER、VEPQQCSIFAAGQY,同位素标记特征肽段VVLV^*^DNADFLK (V^*^, Val-OH-^13^C_5_,^15^N)、DQVV^*^YSLGER、VEPQQCSI^*^(I^*^, Ile-OH-^13^C_6_,^15^N)FAAGQY和侧翼同位素标记特征肽段(内标肽段)DPDGWKVVLV^*^DNADFLKELQ、MADHHKDQVV^*^YSLGERCQPGMG、LPSMCRVEPQQCSI^*^FAAGQY由上海强耀生物科技有限公司合成(纯度≥95%)。3种标准蛋白质由南京金斯瑞生物科技有限公司重组表达获得,并经SDS-PAGE和Western blot验证。甲酸、甲醇、乙腈(色谱级)购自德国默克公司,碳酸氢铵(NH_4_HCO_3_,纯度≥99%)、氯化钠(NaCl,纯度≥99%)、十二烷基硫酸钠(SDS,纯度≥99%)、二硫苏糖醇(DTT,纯度≥99%)、碘乙酰胺(IAA,纯度≥99%),三羟甲基氨基甲烷盐(Tris,纯度≥99%)和牛血清蛋白质(BSA,纯度≥99%)均购自美国Sigma公司;胰蛋白酶(测序级,Promega,美国);赖氨酰基内切酶(Lys-C,质谱级,Wako,日本); BCA蛋白质定量试剂盒(生工生物工程(上海)有限公司)。用于分析的稻米及制品均在线上平台购买。

### 1.2 肽段标准溶液配制

按照生产商提供的肽段溶液配制指导书,分别准确称取1 mg人工合成的特异性肽段、同位素特征肽段和内标肽段用20%乙腈水溶液溶解并依次稀释成一系列浓度的标准储备液。其中,*α*-球蛋白对应的特征肽段VEPQQCSIFAAGQY和同位素特征肽段VEPQQCSI^*^FAAGQY标准溶液经50 μmol/L IAA在37 ℃黑暗条件下烷基化1 h,得到的肽段经C18-SD柱脱盐后,再用20%乙腈水溶液依次稀释成一系列浓度的标准溶液,备用。

### 1.3 样品前处理

1.3.1 蛋白质提取

3种过敏蛋白质的提取参考Satoh等^[11]^和Chen等^[24]^的方法,准确称取0.1 g(精确到0.001 g)稻米及制品粉末于2.0 mL离心管中,加入1.0 mL的蛋白质提取缓冲液(0.5 mol/L NaCl, 30 mmol/L Tris, pH 8.5)于4 ℃恒温混匀振荡仪上振荡提取4 h。将样品置于离心机中以10 000 g在4 ℃下离心10 min后收集上清液后,再加入1.0 mL的蛋白质提取缓冲液重复提取一次,混合两次提取液后,采用BCA蛋白质定量试剂盒测定提取液中的总蛋白质浓度,并贮藏于-80 ℃冰箱中。

1.3.2 蛋白质酶解

在Chiva等^[17]^报道的基础上对酶解体系、水解酶种类和酶量等条件进行比较优化,以期得到最佳酶解效果。最终,实验取0.2 mL蛋白质提取缓冲液于1.5 mL离心管中,并分别加入10 μL内标肽段(5 μmol/L)混匀。加入含0.48 mL 1 g/L SDS的蛋白质变性溶剂(50 mmol/L NH_4_HCO_3_水溶液,pH 8.5)后,经5 μL 1 mol/L DTT在37 ℃条件下还原1 h,再加入25 μL 1 mol/L IAA至终浓度为50 mmol/L,于37 ℃避光反应1 h。按*m*(蛋白质)∶*m*(酶)=20∶1比例依次加入20 μL 0.5 μg/μL Lys-C和Trypsin溶液混匀后置于37 ℃条件下依次酶解4 h和16 h,最后加入适量甲酸终止反应,待净化。

1.3.3 肽段脱盐

依次用甲醇、80%乙腈(含0.1%甲酸)溶液和0.1%(v/v)甲酸溶液活化平衡C18-SD固相萃取小柱后,将酶解肽段混合液加入小柱中,在200 g和4 ℃条件下离心5 min。加入0.5 mL 0.1%甲酸溶液淋洗后,将C18-SD柱放入新15 mL离心管中,依次加入0.5 mL 80%乙腈(含0.1%甲酸)溶液洗脱两次。合并两次洗脱液,过0.2 μm尼龙滤膜后上机分析。

### 1.4 仪器条件

1.4.1 nanoLC-LTQ-Orbitrap条件

色谱条件:色谱柱由Acclaim Pep Map 100(C18, 20 mm×75 μm, 3 μm, Agilent,美国)预柱和Acclaim Pep Map RSLC(C18, 150 mm×50 μm, 2 μm, Agilent,美国)分析柱组成;流动相A为0.1%甲酸水溶液,流动相B为乙腈(含0.1%甲酸)。肽段洗脱条件为:0~120 min, 0~70%B; 120~140 min, 70%B~95%B; 140~160 min, 95%B。进样量为2.0 μL,流速为0.3 μL/min。

质谱条件:纳升离子源喷雾电压为1.8 kV,离子传输管温度为300 ℃,一级质谱利用Orbitrap在*m/z* 300~2000范围内全扫,分辨率设置为60000;将一级质谱扫描获得的TOP10离子用LTQ进行二级碎片离子采集,离子碎裂模式采用碰撞诱导解离(collision-induced dissociation, CID)。

1.4.2 LC-MS/MS条件

色谱柱:Poroshell 120 EC-C18(150 mm×2.1 mm, 2.7 μm, Agilent,美国);柱温:40 ℃,流速:0.2 mL/min;进样量:2.0 μL;流动相由0.1%甲酸水溶液(A)和乙腈(含0.1%甲酸)(B)组成。梯度洗脱程序设置为:0~18 min, 10%B~80%B; 18~24 min, 80%B; 24~24.1 min, 80%B~10%B; 24.1~30 min, 10%B。多反应监测正离子模式采集,离子源温度:450 ℃;喷雾电压:5.5 kV;门帘气压力:130 kPa; Gas 1和Gas 2压力均为250 kPa。

## 2 结果与讨论

### 2.1 特征肽段的鉴定筛选

实验首先利用nanoLC-LTQ-Orbitrap对稻米样品酶解液进行一级全扫描,并结合二级碎片离子,获得实际样品中的肽段指纹图谱。采集数据经Proteome Discover数据库检索软件(ThermoFisher公司,美国)对图谱信息进行匹配检索,最终成功鉴定到3种文献已报道的过敏蛋白质,分别为RAG2、乙二醛酶Ⅰ活性蛋白和α-球蛋白^[11]^,对应的氨基酸序列覆盖率依次为48.2%、59.1%和43.5%,鉴定结果具有较高的可信度(结果见图1)。为确保定量结果的稳定性,实验根据特征肽段的筛选原则^[21,22]^,优先选择响应强度高、氨基酸数目为10~20、*m/z*<1250、无漏切位点、不含易修饰氨基酸的肽段,将DQVVYSLGER[31~40]和VVLVDNADFLK[278~288]分别作为RAG2和乙二醛酶Ⅰ活性蛋白的特征肽段(见图1下划线肽段)。因*α*-球蛋白的氨基酸序列中富含半胱氨酸,酶解产生的肽段中大多含有游离的半胱氨酸。而游离的半胱氨酸在酶解过程中易与IAA发生不可逆的烷基化反应,在质谱鉴定过程中主要以碘乙酰胺加合物的形式存在。因此,实验最终选择鉴定到的VEPQQC(CAM)SIFAAGQY[173~186]作为α-球蛋白的特征肽段。经UniProt和NCBI数据库的基本局部搜索比对工具(BLAST)检索验证特异性,结果表明3个肽段唯一来源于水稻中的RAG2、乙二醛酶Ⅰ活性蛋白和*α*-球蛋白,可作为稻米中3种蛋白质定量的特征肽。

**图1:**
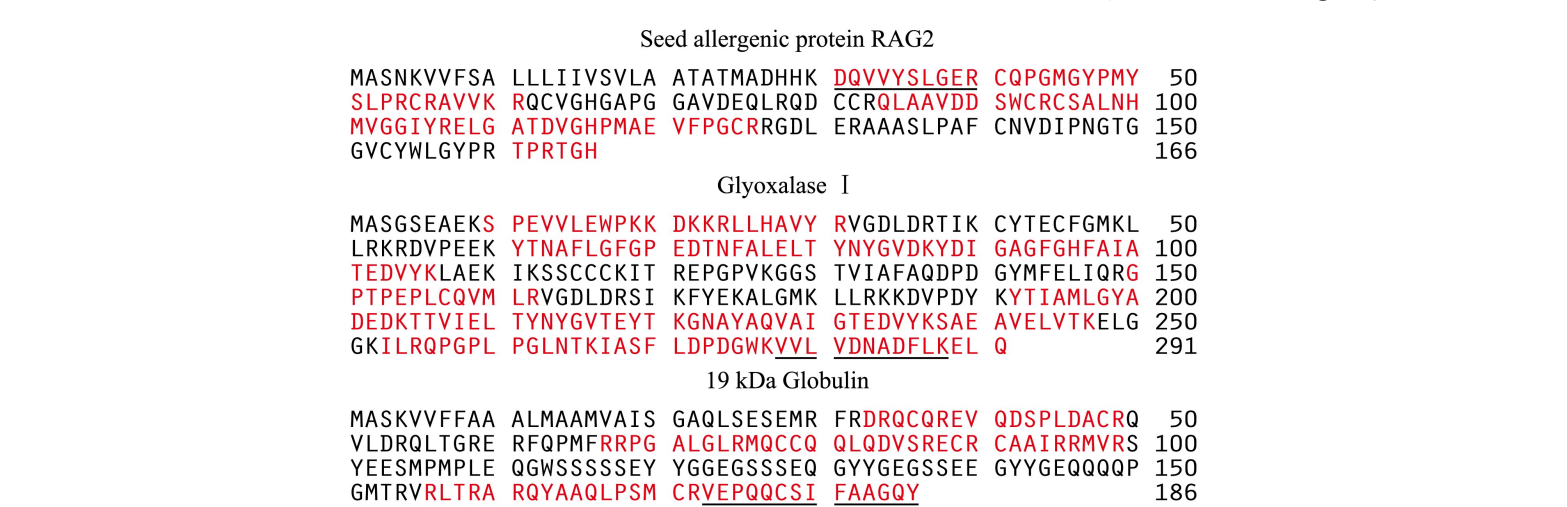
稻米中3种过敏蛋白质的氨基酸序列

### 2.2 特征肽段质谱参数的优化

蛋白质的绝对定量分析主要是采用MRM模式监测特征肽段的碎片离子,实现复杂基质中靶标蛋白质的精确定量。为获得合适的离子对和质谱采集参数,提高检测灵敏度和特异性,本实验采用蠕动泵依次向三重四极杆质谱中引入人工合成的3种多肽单一标准溶液。在正离子模式下进行一级质谱扫描,得到响应强度较高的准分子离子峰[M+2H]^2+^,并进一步采用Skyline软件自动优化碰撞能量,获得灵敏度较高的离子对,3个特异性肽段优化后的离子信息和MRM采集参数见表1。此外,本研究比较了*α*-球蛋白的特征肽段经IAA修饰前后的响应强度差异,结果如图2a和图2b,可以看出,VEPQQCSIFAAGQY的半胱氨酸在与IAA发生烷基化反应后,形成碘乙酰胺加合物,使特征肽段的[M+2H]^2+^增加28.5 Da, 3个碎片离子增加57 Da(见图2c和图2d),离子丰度也显著增强,是未修饰肽段的5倍左右。该结果与Kulevich等^[25]^和王继峰等^[26]^报道的肽段中的半胱氨酸烷基化可提高肽段的离子化效率,进而增强其质谱响应强度的结论相一致。

**表1:** 3种过敏蛋白的特征肽段的离子信息及MRM参数

Uniprot accession	Protein name	Position	Signature peptide sequence	Precursor ion (m/z)	Product ion (m/z)	CE/eV
Q01882	seed allergenic protein RAG2	31-40	DQVVYSLGER	583.5	724.4^a^(y^6+^)	26.3
					823.5(y^7+^)	26.3
					922.6(y^8+^)	24.9
			DQVV^*^YSLGER	586.5	724.5^a^(y^6+^)	26.7
					829.7(y^7+^)	26.6
					928.5(y^8+^)	27.6
Q948T6	glyoxalase I	278-288	VVLVDNADFLK	617.0	822.5^a^(y^7+^)	27.8
					921.6 (y^8+^)	26.9
					1034.0 (y^9+^)	20.0
			VVLV^*^DNADFLK	620.0	822.5^a^(y^7+^)	29.7
					927.7 (y^8+^)	27.1
					1040.0 (y^9+^)	25.9
P29835	19 kDa globulin	173-186	VEPQQC(CAM)SIFAAGQY	799.5	942.5^a^(b^8+^)	24.3
					1089.2 (b^9+^)	24.9
					1160.3 (b^10+^)	24.9
			VEPQQC(CAM)SI^*^FAAGQY	803.0	949.3^a^ (b^8+^)	24.3
					1096.5 (b^9+^)	21.8
					1167.3 (b^10+^)	21.9

* Stable isotope-labeled amino acid. a: quantitative ion. CE: collision energy. CAM: carbamidomethylated.

**图2:**
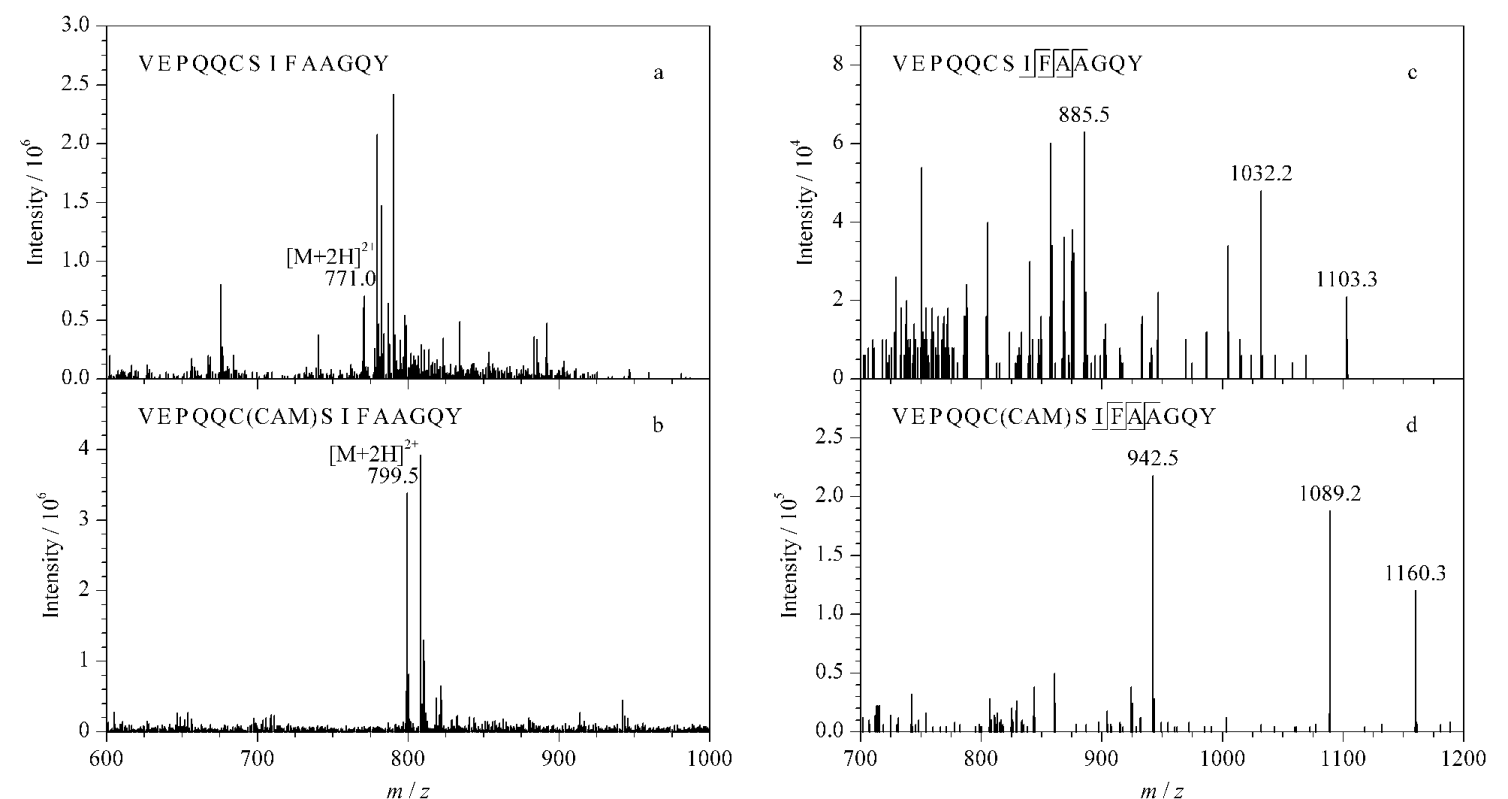
IAA烷基化修饰对*α*-球蛋白特征肽段VEPQQCSIFAAGQY质谱响应的影响

实验对人工合成的3个同位素标记特征肽段的离子信息和质谱参数进行了优化,如表1所示,3个肽段分别在缬氨酸(V)或异亮氨酸(I)的C和N原子进行同位素标记,可使同位素标记肽段的母离子和子离子与未标记肽段之间相差的*m/z*能被低分辨质谱区分。进一步采用MRM正离子模式采集3个特征肽段及同位素特征肽段离子对。结果发现,3种同位素标记肽段各离子对的色谱峰保留时间和离子丰度与未标记特征肽段一致,且色谱峰之间无相互干扰。因此,本实验合成的同位素特征肽段是理想的特征肽段类似物,可用于校正仪器分析误差。

### 2.3 不同酶解条件对酶解效率的影响

目标蛋白质的提取效果和酶解条件在保障定量结果准确性中发挥重要作用^[27]^。鉴于已有文献^[28]^报道了不同溶剂对稻米中过敏蛋白质提取效果的影响,本实验通过合成3种重组蛋白质和侧翼同位素特征肽段,重点比较酶解体系、水解酶种类和酶量,以达到最佳的酶解效果。

2.3.1 酶解体系中表面活性剂的质量浓度

表面活性剂有利于促进蛋白质变性溶解,去除磷脂等非蛋白质杂质的干扰,提高酶解效率。因此,实验向含不同浓度(0、1、5、10、20 g/L)表面活性剂SDS的50 mmol/L NH_4_HCO_3_ (pH 8.5)溶液中分别加入RAG2(120 pmol)、乙二醛酶Ⅰ活性蛋白(60 pmol)和*α*-球蛋白(100 pmol)标准蛋白质,并与相应的侧翼同位素标记特征肽段(100 pmol)共酶解。实验使用内标法定量,获得3种特异性肽段的绝对含量,按特征肽段与相应蛋白质等物质的量比计算目标蛋白质含量。最终以计算得到的3种过敏蛋白质含量与加入的标准蛋白质含量的百分比计算酶解效率,考察酶解体系中表面活性剂对酶解效果的影响。结果如图3a所示,酶解体系中含少量SDS(1 g/L)有利于蛋白质完全变性,可使RAG2、乙二醛酶Ⅰ活性蛋白和*α*-球蛋白的酶解效率分别提高20.6%~35.3%左右。但当SDS的质量浓度超过一定范围时,会抑制Trypsin酶活性,进而降低消化效果。因此,本着获取最佳酶解效率,减少有毒试剂用量的原则,实验最终将含1 g/L SDS的50 mmol/L NH_4_HCO_3_ (pH 8.5)溶液确定为酶解溶剂。

**图3:**
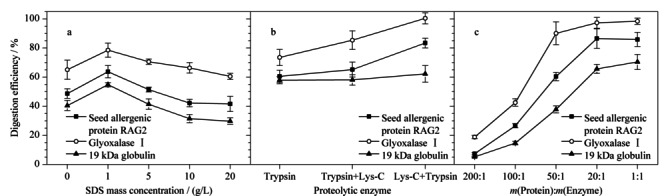
不同酶解条件对3种过敏蛋白酶解效率的影响(*n*=3)

2.3.2 酶种类和酶量

目前常用的蛋白酶主要有胰蛋白酶和赖氨酰基内切酶等。已有研究表明,单一的胰蛋白酶对底物蛋白质酶解不完全,易导致漏切,而Lys-C 与胰蛋白酶互补进行消化,可弥补这些缺点,提高酶解效率和定量准确性。因此,实验分别采用胰蛋白酶酶解20 h、胰蛋白酶酶解4 h后Lys-C酶解16 h、Lys-C酶解4 h后胰蛋白酶酶解16 h 3种方法水解3种标准蛋白质和侧翼内标肽段,内标法定量,考察单一酶解和组合酶解对3种过敏蛋白质定量结果的影响。结果如图3b所示,在Trypsin酶的单独作用下,3种蛋白质的酶解效率为60.5%~73.5%。Lys-C酶主要切割赖氨酸,单独用于蛋白质酶解产生更长的肽链,其酶切效率较低,但Lys-C酶在某些苛刻条件下稳定性强于Trypsin酶。因此,同时采用Trypsin和Lys-C酶切,可显著提高目标蛋白质的酶解效率^[29,30]^,而两种酶不同的酶解顺序也显著影响酶解效果。在Trypsin酶解前使用Lys-C,可使RAG2和乙二醛酶Ⅰ活性蛋白酶解效率提高到83.4%~100.4%。实验结果表明,Lys-C和Trypsin酶组合消化可以互补两者之间的缺点,提高酶解效率,使蛋白质的定量结果更加准确。

实验进一步比较了酶用量(按*m*(蛋白质)∶*m*(酶)=200∶1, 100∶1, 50∶1, 20∶1, 1∶1)对3种蛋白质酶切效果的影响。图3c结果表明,3种蛋白质的酶解效率随着水解酶量添加比例的增加而上升,但当酶量达到20∶1之后,再增加酶量酶解回收率无显著提升。因此,为降低成本,节约试剂,实验最终选择20∶1的加酶比例。

### 2.4 方法验证

本研究通过特异性、基质效应、线性关系、准确度和精密度等多个参数对建立的方法进行验证。

2.4.1 方法的特异性和基质效应评价

实验利用人工合成的3个特征肽段标准品和3种蛋白质水解产生的特异性肽的保留时间来定性。此外,3个特征肽段在MRM模式下同时监测3个离子对,进一步确保了结果特异性。结果表明,3个合成肽段的定性和定量离子保留时间与对应的蛋白质水解肽段保持一致,在连续进样20针后,保留时间的最大偏移在0.5%~1.2%之间。而在未经水解酶消化的样品中,在特征肽段出峰时间范围内无明显峰形。因此,该方法具有较好的特异性。

为评价基质成分对特征肽段的干扰,实验分别用纯溶剂(80%乙腈溶液(含0.1%甲酸))和稻米制品空白酶解液将3种特征肽段配制成5个浓度(1~ 200 nmol/L,每个浓度含50 nmol/L同位素标记特征肽段)的溶液,内标法定量。基质效应评价参考Huang等^[21]^和Chen等^[24]^的方法,以基质效应=(*A/B*-1)×100%计算,其中*A*为以样品基质溶液制作的曲线斜率,*B*为以纯溶剂制作的曲线斜率。结果表明,实验使用内标法时3种特征肽段的基质效应范围在-9.7%~8.5%之间,样品中的基质成分对3种特异性肽段的检测干扰较小。

2.4.2 方法的线性关系、检出限和定量限

将3种特异性肽段配制成一系列浓度的标准工作液,每个浓度含50 nmol/L同位素标记特征肽段,经LC-MS分析。分别以3种肽段的峰面积/内标物峰面积(*y*)和对应浓度比(*x*)绘制标准曲线。表2结果表明,3种肽段在1~200 nmol/L内线性关系良好,相关系数(*r*^2^)均大于0. 9972。以肽段定量离子色谱峰信噪比(*S/N*)为3和10分别为方法的检出限和定量限,对应的蛋白质含量为3种蛋白质的方法LOD和LOQ。结果表明,该方法中3种蛋白质的LOD和LOQ分别为3 mg/kg和10 mg/kg。

**表2:** 3种特异性肽段的线性范围、线性方程、相关系数(*r*^2^)及过敏蛋白质的检出限和定量限

Protein	Signature peptide	Linear range/ (nmol/L)	Linear equation	r^2^	LOD/ (mg/kg)	LOQ/ (mg/kg)
Seed allergenic protein RAG2	DQVVYSLGER	1-200	y=0.817x-0.015	0.9984	3	10
Glyoxalase I	VVLVDNADFLK	1-200	y=0.783x-0.008	0.9991	3	10
19 kDa globulin	VEPQQC(CAM)SIFAAGQY	1-200	y=0.854x-0.011	0.9972	3	10

y: peak area ratio of the analyte to internal standard; x: concentration ratio of analyte to internal standard.

2.4.3 方法的回收率和精密度

实验向空白的稻米制品基质中加入低、中和高水平的3种过敏蛋白质标准品,经1.3.1节步骤提取后,加入3种侧翼同位素标记内标肽段与标准蛋白质共酶解。用内标法计算得到定量肽段浓度后,再按照特异肽段与目标蛋白质的物质的量比为1∶1的比例,计算目标蛋白质的含量。分别将每个浓度水平在一天内连续测定7次和连续测定5天,计算日内精密度(intra-RSD)和日间精密度(inter-RSD)。实验结果如表3所示,本方法3种蛋白质的加标回收率为80.6%~103.7%,日内和日间精密度均小于11.5%。

**表3:** 3种过敏蛋白质在空白稻米制品中3个加标水平的回收率、日内精密度和日间精密度

Protein	Peptide	Spiked level/ (mg/kg)	Recovery/%	Intra-RSD/% (n=7)	Inter-RSD/% (n=5)
Seed allergenic protein RAG2	DQVVYSLGER	10	88.5	5.8	7.9
		50	94.6	4.7	5.4
		200	103.7	1.1	6.1
Glyoxalase I	VVLVDNADFLK	10	83.5	7.1	11.5
		50	94.4	3.5	8.6
		200	91.2	0.4	5.3
19 kDa globulin	VEPQQC(CAM)SIFAAGQY	10	80.6	6.9	10.2
		50	85.2	7.2	7.7
		200	84.9	5.6	6.5

### 2.5 方法应用

为验证本研究建立方法的适用性,实验最后采用建立的HPLC-MS/MS法结合稳定同位素标记肽段方法分析了20份不同稻米及制品中3种过敏蛋白质含量,分析结果见表4。在15份稻米样品中均能检测到3种过敏蛋白质,其含量范围在(0.014±0.001)~(3.55±0.15) mg/kg; 5份稻米制品中有2份样品中检出*α*-球蛋白,其含量为(0.022±0.004)~(0.049±0.005) mg/kg。3种过敏蛋白质的特征肽段在实际样品中的定量离子色谱图见图4。

**表4:** 实际样品中3种过敏蛋白质的含量测定结果

Sample No.	Species	Sample name	Seed allergenic protein RAG2	Glyoxalase I	19 kDa globulin	
1	Indica	Liangyou 421	0.016±0.001	0.031±0.001	2.15±0.08	
2		Zhongjiazao 17	0.018±0.001	0.025±0.002	3.04±0.21	
3		Yongyou 15	0.023±0.002	0.029±0.003	0.86±0.08	
4	Japonica	Zhongdao 1	0.031±0.001	0.018±0.002	2.07±0.15	
5		Nanjing 5055	0.015±0.001	0.062±0.004	1.17±0.07	
6		Jiafengyou 2	0.022±0.002	0.031±0.002	1.41±0.18	
7	red rice	Ganwanxian 33	0.017±0.002	0.023±0.002	1.78±0.09	
8		Nanhong 1	0.042±0.003	0.061±0.005	2.55±0.09	
9		Zhouxian 1211	0.033±0.002	0.033±0.002	0.64±0.03	
10	black rice	Qindao 2	0.016±0.001	0.028±0.001	1.33±0.11	
11		Heibao	0.019±0.001	0.046±0.003	2.60±0.17	
12		Danyou 1	0.025±0.002	0.055±0.004	1.13±0.10	
Sample No.	Species	Sample Name	Seed allergenic protein RAG2	Glyoxalase I	19 kDa globulin
13	glutinous	Xinxiangnuo	0.034±0.002	0.035±0.002	3.55±0.15
14		Shaonuo 45	0.014±0.001	0.026±0.001	1.48±0.12
15		Yunxiangnuo 1	0.021±0.002	0.041±0.003	0.84±0.06
16	rice food product	Product 1	ND	ND	ND
17		Product 2	ND	ND	ND
18		Product 3	ND	ND	0.049±0.005
19		Product 4	ND	ND	0.022±0.004
20		Product 5	ND	ND	ND

ND: not detected.

**图4:**
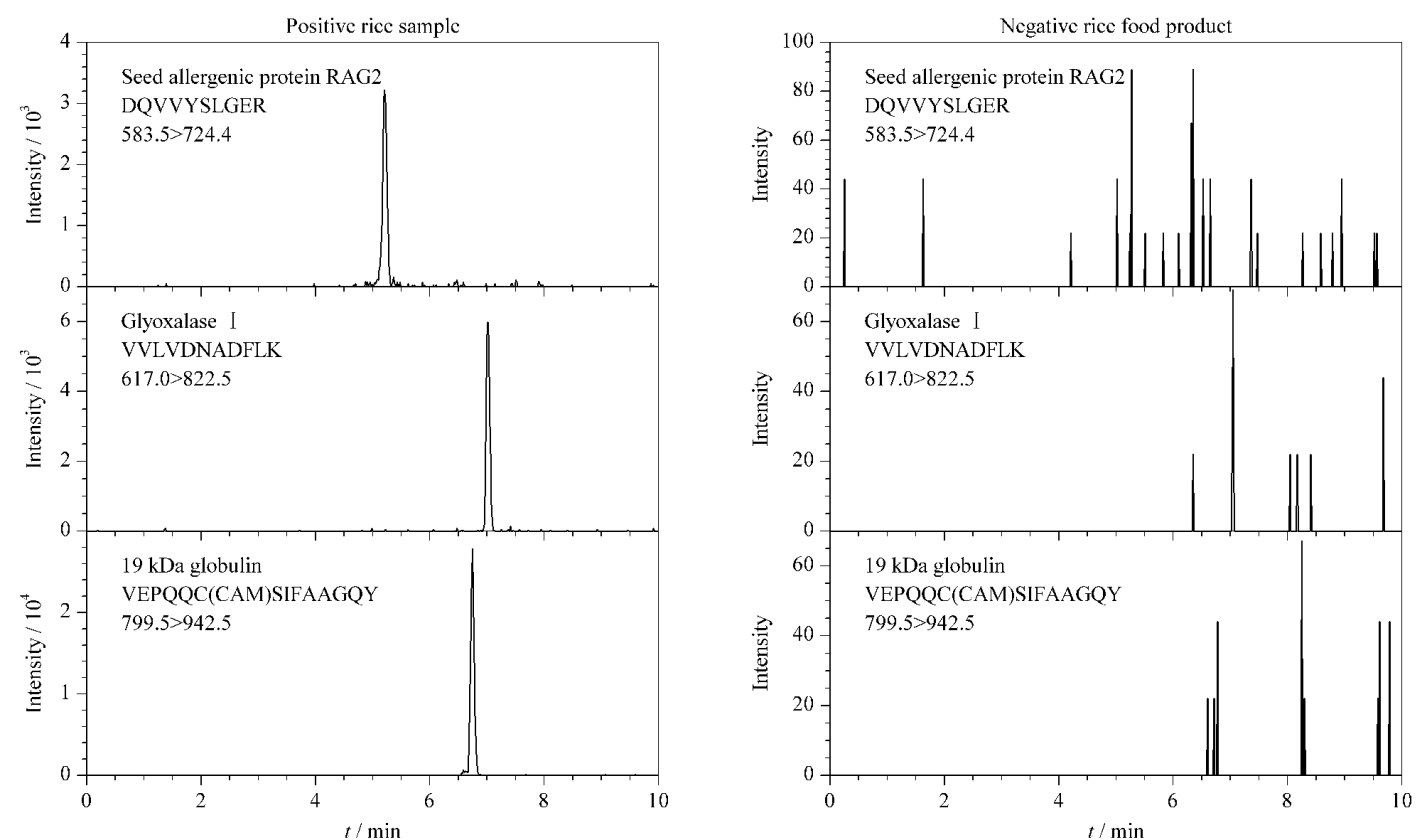
稻米及制品中3种过敏蛋白质特征肽段的定量离子色谱图

## 3 结论

本实验建立了一种同时分析稻米及制品中3种过敏蛋白质含量的检测方法,该方法基于Bottom-up策略,结合LTQ-Orbitrap技术和Protein Discovery软件,成功筛选到稻米中3种过敏蛋白质的特异肽段。通过人工合成特征肽段、同位素内标肽段及蛋白质标准品,优化质谱参数和酶解条件,以3种过敏蛋白质相应特征肽段为分析对象,实现稻米及制品中3种过敏原的同时定量分析。该方法稳定性好,检测灵敏度高,操作简便,适用于各类稻米及制品中3种过敏蛋白质含量的测定,可作为正确评价和标记过敏原的有效工具,以保护人们免受大米过敏原的危害。
